# A Russian Version of the Emotional Autonomy Scale: Primary Adaptation Data

**DOI:** 10.11621/pir.2022.0306

**Published:** 2022-09-30

**Authors:** Tatiana Konshina, Tatiana Sadovnikova

**Affiliations:** a Educational Psychological Service, Haifa, Israel; b Lomonosov Moscow State University, Moscow, Russia

**Keywords:** Adolescence, Personality Autonomy, Emotional Autonomy, Cultural-Historical Theory by L.S. Vygotsky, Child-Parent Relationship, Social Situation of Development, Identity

## Abstract

**Background:**

The formation of emotional autonomy in child-parent relations is one of the main developmental tasks of adolescence (Havighurst, 1972). The theoretical framework of our study comes from the Age-Related Cultural-Historical Approach (Vygotsky, 2000; Leontiev, 1978; Bozhovich, 2009; Elkonin, 1972) and the Theory of Emotional Autonomy Formation by L. Steinberg & S. Silverberg (1986). Here we present the results of a test with the Russian version of the “Emotional Autonomy Scale” (EAS) as validated by L. Steinberg & S. Silverberg (1986).

**Objective:**

We conducted a substantial psychometric analysis of the EAS scales on a Russian sample.

**Design:**

The participants were 222 pupils from the 9th to 11th grades in Moscow schools (age 14–17; M = 15.89; SD = 0.91). A comparative and structural analysis was conducted to review the reliability of the EAS Russian version, administered by the authors.

**Methods:**

The pupils were evaluated with 1) the authors’ Russian version of the EAS by L. Steinberg & S. Silverberg (1986); and 2) the Parent-Child Interaction questionnaire (PCI) by I. Markovskaya (1999).

**Results:**

The fit of Steinberg’s original Four-factor model (L. Steinberg & S. Silverberg, 1986) and of the Beyers’ and colleagues’ Seven-factor model (2005) were studied on a Russian sample for the first time. The Four-factor model was chosen as the final model due to better fit indices and Cronbach’s alpha coefficient. The CFA showed the model fit indices to be acceptable (RMSEA = 0.07) or slightly less than the good fit values (CFI = 0.74). The validity analysis was conducted using the PCI by I. Markovskaya (1999).

**Conclusion:**

The aim of theoretical analysis, approbation, and validation of the EAS on a Russian sample was achieved: the authors’ version of the EAS is a valid and reliable instrument to measure adolescents’ emotional autonomy in a Russian sample.

## Introduction

Modern Russian society is characterized by rapid, visible dynamic social transformations, with low predictability (Akulich & Melnik, 2018; Martsinkovskaya, 2019). Coping with the situation of uncertainty during the COVID-19 period has become the subject of new studies by Russian scientists (Ermolaev, et al., 2021; Sidyacheva & Zotova, 2020; Shaigerova, et al., 2018). Modern world challenges highlight the crucial importance of developing a young person’s personality in a situation of social instability, and helping them become capable of making independent, responsible choices.

Th us, the theoretical and practical significance of the formation of personal autonomy, the central task of development (R. Havighurst) in adolescence, is not in doubt. In modern developmental psychology, adolescence is traditionally characterized as a critical period (Erikson, 1994; Stern & Eichorn, 1989; Prikhozhan & Tolstykh, 2016; Polivanova, 2016). Multiple changes occur in the life of an adolescent child, reflecting the teenagers’ urgent need to take a new position in relations with adults and peers. The problem of self-determination is effectively operationalized within the framework of the Age-Psychological Approach (Vygotsky, 2000; Leontiev, 1978; Bozhovich, 2009; Elkonin, 1972). The relationship between a child and a parent is an important condition affecting the development of an adolescent’s autonomy.

The concept of the social situation of development (SSD) was suggested by L.S. Vygotsky (1956) to define the determinant of a child’s development (Vygotsky, 2000). Vygotsky developed the SSD theory by studying the structure and dynamics of psychological age as a macro unit of development periodization. The notion of SSD has been enriched in the works of his colleagues and followers (Bozhovich, 2009; Elkonin, 1972; Leontiev, 1978; Karabanova, 2010). The contexts for the SSD lie in child-milieu interaction, particularly in the interaction with adults (Karabanova, 2010).

The social situation of development (SSD) of modern Russian adolescents significantly changed in comparison with the SSD of their peers during the 1990s, 2000s, and 2010s (Dubrovina, 2020; Karabanova & Bukhalenkova, 2016; Martsinkovskaya, 2019; Polivanova, 2016; Sobkin & Kalashnikova, 2019). The adolescents’ problems of self-determination in different life areas are related to the features of this psychological age (Vygotsky, 2000; Leontiev, 1978; Bozhovich, 2009; Elkonin, 1972). The empirical studies done in 2000-2010 confirm the complication of the SSD of modern adolescents, as reflected in the intercorrelation between the adolescents’ perception of the nature of success, and their evaluation of own success, their sense of self-esteem (Bukhalenkova & Karabanova, 2018; Konshina & Sadovnikova, 2018; Sobkin & Kalashnikova, 2020), the lengthening of the period of childhood, and infantilism as a common trait representative of modern youth (Martsinkovskaya, 2019; Tolstykh, 2015). The connection between the autonomy of the individual and the motivation for volunteer activities in adolescence is also evident (Molchanov et al., 2022).

In the cohort of modern Russian adolescents there are groups that differ in the level of personal autonomy, opinions about freedom and responsibility, separation from the parental family, career aspirations, and the type of orientation to personal success, etc. (Karabanova & Bukhalenkova, 2016; Lianguzova, et.al., 2018; Malenova & Potapova, 2018; Sadovnikova & Dzukaeva, 2017).

In the Age-Specific Approach, the transformative features of the adolescent-parent relations are considered to be characteristics of the social situation of development. SSD was defined by L.S. Vygotsky as “the unique, specific for a particular age, inimitable relationship between child and social surroundings” (Vygotsky, 2000, p. 903). The structure of the social situation of child’s development includes two components: the first, the objective component, reflects the child’s objective position in the system of socio-cultural expectations, norms, and requirements; the second, the subjective component, is the system of “orientated images” which defines the child’s interaction and cooperation with peers and adults (Karabanova, 2010).

The subjective component is shared by the participants in their communication and interaction. The child builds up his relations with an adult in the process of active orientation (Galperin, 1989; Podolsky, 2012, 2017; Podolsky & Idobaeva, 2014) and on the basis of his personal images in communication and cooperation. Adolescents’ emotional experiences influence the way their development is affected by features of their social surroundings. Their communication with parents and peers and joint activity in different contexts of SSD (family, school, friends, etc.) allow them to realize the different trajectories and patterns of personal autonomy development and individuation in adolescence and youth (Dzukaeva & Sadovnikova, 2014; Kins, et. al., 2013; Litvinova, 2020; Poskrebysheva & Babkina, 2020; Ryan & Lynch, 1986).

The central developmental tasks of adolescence are the formation of an identity, the development of value orientations, the creation of an autonomous morality on the basis of a new level of teenagers’ intellectual opportunities, the development of reflection, and the construction of life plans (Havighurst, 1972). The “main age activity” (Leontiev, 1978) of adolescence is vocational self-determination (Elkonin, 1972; Klimov, 2004; Pryazhnikov, 2007).

There is an objective necessity for parental involvement in the process of modern adolescents’ professional future orientation. This need can be explained by the insufficient development of the adolescent’s autonomy and the need to attract the resources of the parental family under the conditions of educational system modernization in the Russian Federation (Asmolov & Guseltseva, 2019; Karabanova, 2018; Klimov, et al., 2021; Konshina, 2018; Konshina & Sadovnikova, 2018; Molchanov, et al., 2019).

The process of personal autonomy formation in adolescence, and the process of psychological separation from parents, are long and complex processes mediated by child-parent relationships (Dzukaeva & Sadovnikova, 2014; Litvinova, 2020; Malenova & Potapova, 2018; Poskrebysheva & Kremenchustkaya, 2018; Rean, 2017; Thoennissen, et al., 2010; Zimmer-Gembeck & Collins, 2003). They have been widely explored for both early and senior adolescence in Russian psychology at the beginning of the 21st century by Burmenskaya (2005), Pupyreva (2007), Poskrebysheva (2010), Stankovskaya (2014), Leontiev & Sulimina (2015), Dzukaeva (2016), and Molchanov et al., (2017). The concept of personal autonomy has a long history of development and been specified within the framework of various theoretical approaches: the psychoanalytic approach, the epigenetic concept of Erikson (1994), the theory of social learning (Bandura, 1977), the existential-humanistic theory of human motivation of Maslow (1962), Rogers (1959), etc. O.A. Karabanova and N.N. Poskrebysheva emphasize that the term “autonomy” is an umbrella-term: “Its definition is reflected in the existence of different concepts that describe the phenomenology of individual autonomy and of its development in adolescence” (Karabanova & Poskrebysheva, 2013, p. 621).

Modern research shows the importance of emotional autonomy for solving developmental age tasks in late adolescence (Kins et al., 2013; Parra, et al., 2015; Poskrebysheva & Kremenchustkaya, 2018; Poskrebysheva & Babkina, 2020; Puklek & Gril, 2010; Th oennissen et al., 2010).

The separation-individuation process refers to specific developmental challenges in early childhood and adolescence. According to M. Mahler (1977), in early childhood separation-individuation can be considered a “psychological birth” process. The adolescent establishes a sense of individualized self and becomes less psychologically dependent on his parents, disengaging from the relations and representation of parental family that was formed in the infancy period. The concept of “emotional autonomy,” as proposed in the psychodynamic approach of the mid-1980s in the works of L. Steinberg and S. Silverberg, is based on the idea of a “second phase of separation-individuation,” which was suggested by P. Blos (1962, 1967).

Genuine autonomous functioning does not develop until late adolescence and coincides with the development of a coherent sense of personal identity (E. Erikson). L. Steinberg and S. Silverberg define the term “emotional autonomy” as independency from parents, or individuation. The term was meaningfully connected with the concepts of “deidealization of parents” and “changing the image of parents in the ‘eyes’ of a teenager” (Steinberg & Silverberg, 1986). In the 1990s, L. Steinberg with colleagues (1993) clarified the understanding of the emotional component of autonomy, paying particular attention to adolescents’ changed perception of the parental image. The development of the “mature,” realistic, balanced image of parents, coupled with the adolescent’s growing responsibility for his own decisions and values, are considered the basis for the emotional stability and emotional autonomy of the adolescent’s personality. L. Steinberg’s colleagues, S. Silverberg and M. Baltes, developed the concept of autonomy as the achievement of self-confidence, and the increasing ability for self-regulation, related, among other things, to the learning process and behavioral aspects (Baltes & Silverberg, 1994). The authors also include in the concept of autonomy a person’s own initiative, self-guidance, and independence, contrasting these personality traits with the propensity to obey, an obedience to “external” rules and authorities.

The “Emotional Autonomy Scale” (EAS) questionnaire was created based on this theory. The questionnaire operationalizes two cognitive components — “Parental Deidealization” and “Perceives Parents as People” — and two affective components — “Nondependency on Parents” and “Individuation” (Steinberg & Silverberg, 1986). The EAS permits us to assess features of emotional autonomy from the parents as the adolescents perceive them.

The development of emotional autonomy is an important line of psychological development in adolescence. The task of clarifying the theoretical construct and the need to adapt foreign methods for a Russian-language sample remain relevant (Beyers, et al., 2003; Dergacheva & Leontiev, 2011; Dozortseva & Burykina, 2016; Dundarova, 2008; Poskrebysheva & Babkina, 2020). The EAS is widely used by researchers all over the world. T. Fuhrman & G. Holmbeck investigated the relationship between emotional autonomy and adolescents’ adjustment as moderated by several individual, familial, and cultural contexts. Their study showed the positive association between emotional autonomy and adolescent adjustment in cases of a more stressful family environment. The findings suggested that higher scores of emotional detachment from parents on the EAS index are detrimental in supportive familial environments but adaptive in less supportive familial environments (Fuhrman & Holmbeck, 1995).

A study of Indian adolescents organized by S. Tung & D. Sandhu showed significant positive correlations between all dimensions of emotional autonomy and wellbeing in adolescence. The “healthy” identity statuses of achievement and moratorium in the adolescent period also were positively correlated with emotional autonomy (Tung & Sandhu, 2005).

When studying the main family factors for the development of autonomy and separation processes in adolescence, Poskrebysheva and Babkina (2020) used three questionnaires: 1) the well-known SITA questionnaire developed by J.B. Levine with colleagues (Levine et al., 1986); 2) the “Autonomy questionnaire” for studying the autonomy of adolescents by N.N. Poskrebysheva and O.A. Karabanova (2010); and 3) the translation of the EAS into Russian proposed by the authors of this article in our earlier work (Konshina & Sadovnikova, 2018).

Few researchers have examined the factor structure of the EAS. The cross-cultural study of M. Schmitz & J. Baer (2001) showed that the EAS exhibited poor construct validity and behaved quite differently for different grades (6, 8 and 10) and different ethnic groups (African American, European American, and Mexican American). M. Schmitz & J. Baer offered to reexamine the conceptual foundations of emotional autonomy and to develop better measures of those concepts for adolescents (Schmitz & Baer, 2001). In later research W. Beyers and colleagues also showed the lack of construct validity of the existing EAS factor structures on a Belgian sample of adolescents. The scientists suggested a model with seven first-order factors (Deidealization, Nondependency, Non-imitation, Privacy, Perceived Ignorance, Distrust, and Perceived Alienation) and two second-order factors (Separation and Detachment) that proved invariant and equal across gender and grade (Beyers et al., 2005).

Modern Russian psychology has a lack of instruments for measuring autonomy. In spite of this, the issue of the autonomy development is widely studied by Russian researchers (Kharlamenkova et al., 2015; Karabanova & Poskrebysheva, 2013; Dzukaeva & Sadovnikova, 2014; Molchanov, Almazova, Zapunidi, & Poskrebysheva, 2017). Few questionnaires contain “autonomy” subscales (for example, the Russian version of ADOR questionnaire by Wasserman, Gor’kovaya, & Romytsina (2001), and the Parent-Child Interaction questionnaire by I. Markovskaya (Markovskaya, 1999). The “Autonomy questionnaire” was developed by N. Poskrebysheva & O. Karabanova in 2010 as a new method to research four aspects of adolescents’ autonomy. The study also contained the Russian version of the EAS. In this research, correlation analysis of the EAS Russian version didn’t show significant correlations with valid Russian questionnaires — the ADOR (Adolescents about Parents) by Wasserman, Gor’kovaya, and Romitsyna (2001) and the PCI (Parent-Child Interaction), by I. Markovskaya (1999).

The use of structural analysis to build a factor model has spread widely in the social sciences world (Loehlin, 1998; Hu & Bentler, 1999; Jackson, et al., 2009; Hooper, et al., 2008). Recently this method has also been used extensively among Russian psychologists (Ostapenko, 2013; Krichevec et al., 2018).

*The general objective* of our research was to design tools in Russian that would expand the understanding of the development of emotional autonomy from parents in older adolescence.

The specific objectives were to improve the Emotional Autonomy Scale (Steinberg & Silverberg, 1986) structure on a Russian sample and to validate it using the Parent-Child Interaction Questionnaire (Markovskaya, 1999).

In Russian developmental psychology, the child-adult relationship system is an object of development (Vygotsky, 2000; Venger, et al., 1988), which perfectly meets the goals of our research.

## Method

### Participants

The sample consisted of 222 pupils from 9th to 11th grades in Moscow schools (Moscow, Russia): 125 girls (56.3%) (M = 15.96; SD = 0.83) and 97 boys (43.7%) (M = 15.84; SD = 0.86) of ages 14–17 (M = 15.89; SD = 0.91).

The study was conducted on the basis of the principles of voluntary participation, anonymity, and confidentiality. Adolescents were informed about the study protocol beforehand. The teenagers were given the opportunity to meet individually to discuss individual outcomes. Parents of the adolescents were informed of the study design and signed informed consent.

### Procedure

Participants completed the Russian version of Emotional Autonomy Scale (Konshina, 2018). The pupils filled out the questionnaire in groups of 20 to 30 persons according to school grades during normal school time. The EAS questionnaire was one of the battery of techniques suggested to study different aspects of interaction with parents in late adolescence.

#### Design

Comparative and structural analysis was conducted by the authors to review the reliability of EAS Russian version. The methods used were the authors’ Russian version of “Emotional Autonomy Scale” by L. Steinberg & S. Silverberg (1986) and “Parental-Child Interaction Questionnaire” (PCI) (Markovskaya, 1999).

### Questionnaires

#### Emotional Autonomy Scale

The EAS is composed of four subscales: “Deidealization of Parents” (5 items) and “Parents As People” (6 items) — the two cognitive components of EA); and “Non-dependency on Parents” (4 items) and “Individuation” (5 items) — the two affective components of EA. The 20 items of the EAS were rated on the 4-point Likert-type scale that was used in the original EAS procedure suggested by L. Steinberg & S. Silver berg. The scale contained four points, from 1 — “strongly disagree” to 4 — “strongly agree.”

#### Parental-Child Interaction

The “Parental-Child Interaction” questionnaire (PCI) was developed by I. Markovskaya as an instrument to describe aspects of parent-child interaction (Markovskaya, 1999). The questionnaire contains 60 items distributed among 10 subscales: “Demanding,” “Strictness,” “Autonomy — Control,” “Emotional distance — Intimacy,” “Rejection — Acceptance,” “Cooperation,” “Disagreement — Compliance,” “Inconsistency — Sequence,” “Authority of the Parent,” and “Satisfaction with the Relationship.” The questionnaire was presented in two variants —first, for the adolescent’s perception of relations with the mother and, second, for the adolescent’s perception of relations with the father.

The PCI was used for validation of the EAS on the Russian sample as a sound and secure method reflecting the main aspects of the child-parent relationship in adolescence.

## Results

The results were statistically analyzed with IBM SPSS program, ver. 21.0. The factor analysis was built using the EQS program, ver. 6.2, and the structural model was built using AMOS program, ver. 23.0.

### The reliability measures

The first step of our study was a comparative analysis of EAS reliability in light of previous research.

The findings of the research organized in 2016–2017 were compared with the results of the original verified EAS (Steinberg & Silverberg, 1986). The internal consistency of the EAS measure of both studies is presented in *Table 1.*

**Table 1:** Cronbach’s alpha of EAS measure

Scales of EAS / Research	Parents as People (6 items)	Parental Deidealization (5 items)	Nondependency on Parents (4 items)	Individuation (5 items)	EAS, total
Steinberg & Silverberg, 1986	.61	.63	.51	.60	.75
Konshina & Sadovnikova, 2016–2017	.60	.55	.48	.55	.73

The Cronbach’s alpha of the EAS Russian version in the 2016-2017 research was a little less than in the original Steinberg & Silverberg research (Steinberg & Silverberg, 1986) over all subscales. The general measure was characterized by pretty high reliability (Cronbach’s alpha = .73), but three of four subscales had a reliability coefficient between .47 and .60 (*Table 1*). Such a result is considered to be acceptable.

The sample size of the study allowed the use of CFA (Krichevec, et al., 2020; Beyers, et al., 2003).

The latest factor research of the EAS was organized in 2005 by W. Beyer and colleagues on a Belgian sample. Beyers’ research (2005) showed better functioning with the seven-factor model than the four-factor model. We compare Beyers’ results on the Belgian sample and our findings on a Russian sample in *Table 2.*

**Table 2:** Fit indices for the Four-factor and Seven-factor models — Beyers and colleagues’ research, 2005, and author’s research, 2016–2017

Model description	Research	χ^2^(df)	RMSEA	SRMR	CFI	CAIC
Four-factor model	Beyers 2005 et al.,	2155.47 (164)	.069	.070	.91	2561.98
	Konshina & Sadovnikova, 2016–2017	309.25 (222)	.070	.092	.74	708.21
Seven-model factor	Beyers 2005 et al.,	884.92 (149)	.044	.049	.96	1423.9
	Konshina & Sadovnikova, 2016–2017	603.81 (163)	.122	.119	.70	491

As can be seen in the table, the four-factor model suggested by L. Steinberg & S. Silverberg (1986) showed better results for the Russian sample than the seven-factor model suggested by W. Beyers and colleagues. The lower RMSEA and SRMR values and higher CFI indicated better fit.

To establish the suitability of the models, the recommended criteria were used: CFI > .90, RMSEA < .08, SRMR < .08 (Kline, 2011; van de Schoot, et al., 2012). It is known that the best solution is determined by a combination of these parameters. Let’s consider the results we obtained.

Lower RMSEA and SRMR values and higher CFI indicate better fit (Loehlin, 1998; Hu & Bentler, 1999). The reliability of the model still couldn’t be valued as high (RMSEA > 0.5; and CFI < 0.9) but can be considered passable (RMSEA ≤ 0.07; CFI > 0.7).

You can see that the indicators of our model are close to Hu and Bentler’s Two-Index Presentation Strategy (1999), where Combinational Rules call for an RMSEA of 0.06 or lower and an SRMR of 0.09 or lower (RMSEA = 0.07; SRMR = 0.092).

The purpose of the S. Cangur & I. Ercan study (2015) was to investigate the impact of estimation techniques and sample sizes on model fit indices in structural equation models constructed according to the number of exogenous latent variables under multivariate normality. It has been shown that the findings of various authors, except for the RMSEA, were quite different from the study results of X. Fan and E.A. Sivo (2007). In addition, S. Cangur & I. Ercan (2015) referred to the work of E.E. Rigdon (1996), who emphasized the need to use RMSEA with large sample sizes and research in which RMSEA and CFI were compared. In CFA results, the model fit indices were acceptable (RMSEA = 0.07) or slightly less than good fit values (CFI = 0.74).

The factor analysis showed that few items influenced more than one subscale, and one item (item 19) could be expected to raise the model reliability (*Table 3*).

**Table 3:** The significant correlations between EAS and PCI (mother’s variant) subscales.

Subscales EAS\ PCI	ED–I	R–A	C	I-S	AP	SR
Deidealization	–.468**	–.346**	–.305**	–.206*	–.622**	–.495**
Parents As People	–	–	–	–.271**	–	–.197*
Nondependency	–.355**	–.276**	–.326**	–	–.419**	–.353**
Individuation	–.458**	–.505**	–.394**	–.471**	–.524**	–.485**

Note: ED–I = Emotional Distance — Intimacy; R–A = Rejection — Acceptance; C = Cooperation; I–S = Inconsistency — Sequence; AP = Authority of the Parent; SR = Satisfaction with the Relationship. * —p < .05, ** —p < .001

#### The Validation

The next step of our research was to validate the EAS Russian version using an already validated and secure Russian questionnaire.

One instrument most closely related in topic and by instruments used by Russian researchers was the “Parent-Child Interaction” (PCI) questionnaire (Markovskaya, 1999). Adolescents filled out the questionnaire in two variants — one for interactions with the mother and the other for interactions with the father.

### The Interrelation of autonomy development with the child-parent relations of adolescents

Generally, the PCI items are close by sense to the EAS items.

According to the study findings, adolescents’ emotional autonomy components (in relation with mother) negatively correlated (all the correlations mentioned in the present article are significant) with the parameters of child-parent relations (*Table 3*). There was a negative interrelationship between “Deidealization” (EAS) and, respectively, “Emotional Distance — Intimacy” (PCI) (r = –0.468), “Rejection — Acceptance” (PCI) (r = –0.346), “Cooperation” (PCI) (r = –0.305), “Inconsistency — Sequence” (PCI) (r = –0.206), “Authority of the Parent” (PCI) (r = –0.622), and “Satisfaction with the Relationship” (PCI) (r = –0.495). There was a negative interrelationship between “Parents As People” (EAS) and, respectively, “Inconsistency — Sequence” (PCI) (r = –0.271), and “Satisfaction with the Relationship” (PCI) (r = –0.197). There also was a negative interrelationship between “Nondependency” (EAS) and, respectively, “Emotional Distance — Intimacy” (PCI) (r = –0.355), “Rejection — Acceptance” (PCI) (r = –0.276), “Cooperation” (PCI) (r = –0.326), “Authority of the Parent” (PCI) (r = –0.419), and “Satisfaction with the Relationship” (PCI) (r = –0.353).

There was a negative interrelationship between “Individuation” (EAS) and, respectively, “Emotional Distance — Intimacy” (PCI) (r = –0.458), “Rejection — Acceptance” (PCI) (r = –0.505), “Cooperation” (PCI) (r = –0.394), “Inconsistency — Sequence” (PCI) (r = –0.471), “Authority of the Parent” (PCI) (r = –0.524), and “Satisfaction with the Relationship” (PCI) (r = –0.485).

In answering the questions about their relationship with their fathers, the adolescents showed a little different result: more subscales of the PCI were significantly correlated with the EAS subscales. Two of them showed positive correlations: the subscale “Parents as People” correlated with the subscale “Demanding” (r = 0.242), and the subscale “Deidealization” correlated with the subscale “Strictness” (r = 0.242). The remaining EAS scales had negative associations with the PCI questionnaire (father version) (*Table 4*).

**Table 4:** The significant correlations between EAS and PCI (father’s variant) subscales.

Subscales EAS\ PCI	D	S	ED–I	R–A	C	D–C	I–S	AP	SR
Deidealization	–	.242*	–.525**	–.417**	–.425**	–.228*	–	–.628**	–.599**
Parents As People	.242*	–	–	–	–	–	–.411**	–	–
Nondependency	–	–	–.275**	–.291**	–.268**	–	–	–.324**	–.292**
Individuation	–	–	–.311**	–.409**	–.337**	––.216*	–.385**	–.283**	–.305**

Note: D = Demanding; S = Strictness; ED–I = Emotional distance — Intimacy; R–A = Rejection — Acceptance; C = Cooperation; D–C = Disagreement — Compliance; I–S = Inconsistency — Sequence; AP = Authority of the Parent; SR = Satisfaction with the Relationship. * —p < .05, ** = p < .001

In other words, our data allowed us to assume the more difficult nature of the links between indicators of the teenagers’ emotional autonomy and the features of their relations with their fathers, in comparison with the one with their mothers. Such data corresponds to the results of research on teenagers’ separation from their parents done earlier on a Russian sample by T. Syt’ko (2014), V.P. Dzukaeva (2016), and O.V. Sulimina (2016).

## Discussion

Higher levels of PCI estimates mean closer and more intimate relations with one’s parents (the “Emotional Distance — Intimacy,” “Rejection — Acceptance,” “Cooperation,” “Inconsistency — Sequence,” “Disagreement — Compliance,” and “Satisfaction with the Relationship” subscales) and the perception of more controlling parental behavior (“Demanding,” “Strictness”, “Autonomy — Control,” and “Authority of the Parent” subscales). As for the EAS, higher levels of estimates show higher emotional autonomy in four aspects of Parent-Child interaction (the “Deidealization of Parents,” “Perceives Parents As People,” “Nondependency on Parents,” and “Individuation” subscales).

The results show that the predictions were borne out. Those EAS subscales significantly correlated with the PCI subscales.

The “Demanding,” “Strictness,” “Autonomy — Control,” “Rejection-Acceptance,” and “Disagreement-Compliance” subscales of PCI (adolescents’ perception of their interactions with the mother) didn’t show a significant correlation with any of the EAS subscales.

The results reflect, in our opinion, the nonlinear nature of the association between indicators of teenagers’ emotional autonomy, on the one hand, and the parameters of the child-parent relationship, on the other.

However, some researchers consider the correlation analysis data as supportive of the emotional autonomy formation hypothesis (Beyers, & Goossens, 1999; Collins, & Laursen, 2004; Parra, Oliva, & Sanchez-Queija, 2015).

Our results show strong negative correlations between EAS and PCI subscales. We can assume that super-autonomous adolescents perceive their relationships with their mothers as more emotionally distant, characterized by more rejection, less cooperation, and more inconsistency. The adolescents with a high emotional autonomy level considered their mothers’ authority to be low and were less satisfied with their relationship with her. These findings are quite predictable and can illustrate the development of autonomy in relations with the mother among adolescents (Collins, & Laursen, 2004).

V. P. Dzukaeva’s thesis (2016), written under the leadership of T.Yu. Sadovnikova — *i.e.,* that the father and mother play different roles in the teenagers’ autonomy formation — has been verified.

In the traditional Russian family, the father figure is perceived as strict and authoritative. Such results can be explained by the phenomenon of adolescents ceasing to perceive their father figure as an authority and now perceiving him as a strict and demanding member of the family. All the following PCI subscales showed negative correlations with the EAS subscales. We can assume, although only for some teenagers, that super-autonomous adolescents perceive their relationships with their father as more emotionally distant and conflict-ridden , and characterized by more rejection, less cooperation, and more inconsistency. The adolescents with the highest emotional autonomy levels considered the authority of their fathers as low, and those teenagers were less satisfied with their relations with their fathers.

The high predictability of the results can be explained by traditional features of adolescence. As was shown, the Russian version of EAS strongly correlates with the CPI questionnaire.

The results can be accepted as a successful approbation and validation of the EAS on a Russian sample.

## Conclusion

The Russian version of the EAS was validated on a sample of 222 pupils from different Moscow schools. (Beyers et al., 2005; Fuhrman & Holmbeck, 1995; Schmitz & Baer, 2001; Steinberg & Silverberg, 1986).

This study aimed to create a model that would fit Russian realities. By means of analysis, the authors compared the original model fit (Steinberg, & Silverberg, 1986) to the latest model fit suggested in the W. Beyers and colleagues’ research (Beyers et al., 2005). The results showed a better fit for the original four-factor model: Cronbach’s alpha for the EAS = 0.73: for cognitive components (Parental Deidealization and Perceives Parents as People) alpha = 0.55–0.60; and for affective components (Nondependency on Parents and Individuation) alpha = 0.48–0.55. The confirmatory factor analysis showed passable fit indices. In general, the reliability of the Russian EAS version is acceptable.

External validity was checked using the PCI Russian questionnaire, and it was shown to be valid and secure. The correlation analysis showed strong correlations between the EAS and PCI subscales. There were indications of a nonlinear nature of the links between indicators of the teenagers’ emotional autonomy and the features of their relationships with their parents. The super-autonomous adolescents tended to perceive their relations with their mothers and fathers as more distant and characterized by less agreement. The protest against the father’s authority was expressed among super-autonomous teenagers as the perception of the father as strict and demanding. The results were consistent with the theoretical framework of features of the adolescent period. The informative aspects of the correlations can be described by the adolescent age features in the conception of the Age-Related Cultural-Historical Approach.

The Russian version of the Emotional Autonomy scale can be used as a diagnostic method among practicing psychologists in work with teenagers and their families.

This methodology opens up new opportunities for empirical research in the field of developmental psychology and, in a broader context, in the field of personality psychology and psychological well-being. The technique can also be used to solve practical problems of psychological diagnosis, counseling, and psychotherapy in adolescence.

## Limitations

The development of emotional autonomy in the child-parent relationship is an important part of adolescent psychological development. The expansion of the Russian methods to measure emotional autonomy will open up to scientists a new way to research this important stage of aging.

The first steps of EAS validation on a Russian sample were realized.

One limitation of the study was the sample size. One of the ways of improving the validation would be expanding the sample in future research.

Another limitation was the nature of the sample used: students from several schools in Moscow, a megalopolis city. We consider expanding participation to include students from more schools in Moscow, as well as their peers from cities and settlements from other regions (not only megacities), as an important task of further research.

Another limitation was the fact that the fit indices were acceptable, but not perfect. The factor model can be reviewed considering the features of Russian sample.

## Appendix

*The Russian version of Emotional Autonomy Scale*

*(Следующие вопросы будут касаться Ваших родителей. Отметьте степень согласия со следующими утверждениями:)*

**Table:**

Items of the Russian version of Emotional Autonomy Scale (Original Items of the Classic Emotional Autonomy Scale (Steinberg & Silverberg, 1986))	Совер- шенно не сог- ласен	Скорее не сог- ласен	Скорее согласен	Совершенно согласен
1. Я и мои родители соглашаемся во всём (My parents and I agree on everything)				
2. Я обращаюсь к родителям за помощью перед тем, как попытаться решить проблему самостоятельно (I go to my parents for help before trying to solve a problem myself)				
3. Мне всегда было интересно, как мои родители ведут себя, когда я не рядом с ними (I have often wondered how my parents act when I’m not around)				
4. Даже когда мы с родителями расходимся во взглядах, они всегда правы (Even when my parents and I disagree, my parents are always right)				
5. Для подростка лучше обратиться за советом по поводу некоторых вещей к лучшему другу, чем к родителям (It’s better for kids to go to their best friend than to their parents for advice on some things)				
6. Если я сделал(а) что-то не так, моим родителям приходится исправлять это за мной (When I’ve done something wrong, I depend on my parents to straighten things out for me)				
7. Есть некоторые вещи, которые мои родители обо мне не знают (There are some things about me that my parents don’t know)				
8. Мои родители ведут себя со своими родителями по-другому, чем когда они дома со мной (My parents act differently when they are with their own parents from the way they do at home)				
9. Мои родители знают обо мне всё (My parents know everything there is to know about me)				
10. Вероятно, я буду удивлен(а), увидев, как мои родители ведут себя на вечеринке (I might be surprised to see how my parents act at a party)				
11. Я стараюсь придерживаться тех же взглядов, что и мои родители (I try to have the same opinions as my parents)				
12. Мои родители ведут себя на работе так же, как и дома (When they are at work, my parents act pretty much the same way they do when they are at home)				
13. Если у меня возникнет проблема с другом, я обсужу это с мамой или отцом перед тем, как приму решение, что с этим делать (If I was having a problem with one of my friends, I would discuss it with my mother or father before deciding what to do about it)				
14. Мои родители были бы удивлены, увидев, какой(ая) я, когда я не с ними (My parents would be surprised to know what I’m like when I’m not with them)				
15. Когда я стану родителем, я буду воспитывать своих детей именно так, как мои родители воспитали меня (When I become a parent, I’m going to treat my children in exactly the same way that my parents have treated me)				
16. Мои родители, вероятно, говорят о разных вещах, когда я рядом, и когда меня нет поблизости (My parents probably talk about different things when I am around from what they talk about when I’m not)				
17. Есть вещи, которые я буду делать иначе, чем моя мать и отец, когда я сам буду родителем (There are things that I will do differently from my mother and father when I become a parent)				
18. Мои родители вряд ли когда-либо ошибаются (My parents hardly ever make mistakes)				
19. Я хотел(а) бы, чтобы мои родители поняли, кто я на самом деле (I wish my parents would understand who I really am)				
20. Мои родители ведут себя одинаково со своими друзьями и дома со мной (My parents act pretty much the same way when they are with their friends as they do when they are at home with me)				
